# Preparation of One-Emission Nitrogen-Fluorine-Doped Carbon Quantum Dots and Their Applications in Environmental Water Samples and Living Cells for ClO^−^ Detection and Imaging

**DOI:** 10.1155/2023/7515979

**Published:** 2023-04-25

**Authors:** Qianchun Zhang, Haijiang Du, Siqi Xie, Fengling Tian, Xixi Long, Shan Liu, Yun Wu

**Affiliations:** School of Biology and Chemistry, Key Laboratory for Analytical Science of Food and Environment Pollution of Qian Xi Nan, Minzu Normal University of Xingyi, Xingyi 562400, China

## Abstract

Hypochlorite (ClO^−^) has received extensive attention owing to its significant roles in the immune defense and pathogenesis of numerous diseases. However, excessive or misplaced production of ClO^−^ may pose certain diseases. Thus, to determine its biological functions in depth, ClO^−^ should be tested in biosystems. In this study, a facile, one-pot synthesis of nitrogen-fluorine-doped carbon quantum dots (N, F-CDs) was developed using ammonium citrate tribasic, L-alanine, and ammonium fluoride as raw materials under hydrothermal conditions. The prepared N, F-CDs demonstrate not only strong blue fluorescence emission with a high fluorescence quantum yield (26.3%) but also a small particle size of approximately 2.9 nm, as well as excellent water solubility and biocompatibility. Meanwhile, the as-prepared N, F-CDs exhibit good performance in the highly selective and sensitive detection of ClO^−^. Thus, a wide concentration response range of 0–600 *μ*M with a low limit of detection (0.75 *μ*M) was favorably obtained for the N, F-CDs. Based on the excellent fluorescence stability, excellent water solubility, and low cell toxicity, the practicality and viability of the fluorescent composites were also successfully verified via detecting ClO^−^ in water samples and living RAW 264.7 cells. The proposed probe is expected to provide a new approach for detecting ClO^−^ in other organelles.

## 1. Introduction

Sodium hypochlorite at concentrations between 10^−5^ and 10^−2^ mol/L is widely used as a household bleach and disinfectant [1, 2]. ClO^−^ is an important type of reactive oxygen species that is widely used in the antimicrobial immunity of living systems [3, 4]. It is extensively used in daily life as disinfectant and household cleaning agent for water treatment [5]. However, an excessive level of reactive oxygen species may cause certain pathological problems such as tissue aging, chronic inflammatory diseases, and bladder cancer [4, 6]. Therefore, an effective method detecting ClO^−^ in environmental water samples and living cells should be established.

Currently, various approaches have been developed to detect and quantify hypochlorite, such as coulometric [7], chemiluminescence [8], potentiometric [9], and colorimetric methods [10]. Although these methods offer unique advantages, they still possess several limitations, including special equipment, tedious operation, complicated sample preparation, and long time. Compared with other analytical methods, fluorescent probes offer inherent benefits due to their high sensitivity [11], excellent specificity [12], simple manipulation [13, 14], low cost, short analytical times [15], and deep bioimaging capacity [16], which benefit application strategies for in vitro assays and in vivo imaging studies. For example, McCarroll [17] reported a pH-dependent probe to detect HOCl. Furthermore, certain HOCl probes can monitor HOCl in living cells. Duan et al. [18] proposed a hepatoma-specific probe for examining HOCl, and Yuan et al. [19] developed two-photon fluorescent probes for HOCl imaging in mitochondria and lysosomes. Although the precursors play a vital role in improving the quantum yield and property of CDs, the researchers reported many precursors and prepared different CDs to detect ClO^−^ and perform imaging in vitro and vivo [18–20]. Methods for endogenous ClO^−^ detection and measurement in RAW 264.7 cells using nitrogen-fluorine-doped carbon quantum dots (N, F-CDs) are few, and it is necessary to hunt for the precursors and develop the sensitive and efficient sensors. Therefore, designing efficient carbon quantum dots for quantitative analysis of ClO^−^ and selective imaging of ClO^−^ in RAW 264.7 cells is of great significance.

Here, N, F-CDs were simply prepared utilizing the novel precursor via one-pot hydrothermal strategy. The as-prepared composites displayed excellent fluorescence properties, good water solubility, low toxicity, and biocompatibility owing to nitrogen and fluorine doping. Additionally, the N, F-CDs exhibited excellent selectivity and high sensitivity for ClO^−^ with effective fluorescence quenching based on their unique performance. Thus, we successfully developed an effective strategy to quantitatively analyze ClO^−^ in real water. In addition, this probe was seamlessly used for the imaging of living cells and sensitive detection of endogenous ClO^−^ in living RAW 264.7 cells.

## 2. Experimental

### 2.1. Materials

L-Alanine and lipopolysaccharides (LPS) were purchased from J&K Scientific Co. Ltd. (Hebei, China). Ammonium citrate tribasic, sodium hydroxide, sodium chloride, barium chloride, sodium hypochlorite solution, and K_2_Cr_2_O_7_ were supplied by Chongqing East Chuandong Chemical Co. Ltd. (Chongqing, China). Ammonium fluoride and potassium iodide were provided by Xilong Scientific Co. Ltd. (Shantou, China). Quinine sulfate, L(+)-ascorbic, cupric chloride, dibasic sodium phosphate, potassium dihydrogen phosphate, sodium bicarbonate, calcium chloride, manganese sulfate, potassium chloride, L-cysteine, phorbol 12-myristate 13-acetate (PMA), hydrogen chloride (36.5%, w/w), BaCl_2_·2H_2_O, MgCl_2_, ZnCl_2_, K_2_S_2_O_8_, H_2_O_2_, K_2_SO_3_, and KMnO_4_ were sourced from Aladdin Chemistry Co. Ltd. (Shanghai, China). Ultrapure water was used in all experiments.

### 2.2. Instrumentation and Characterization

A Shimadzu RF-6000 spectrometer (Tokyo, Japan) was used for fluorescence intensity measurements. A UV-5500 spectrophotometer (Shanghai Metash Instruments Co. Ltd., China) recorded the UV-vis absorption spectra at 20°C. The particle size of N, F-CDs was accurately measured using Tecnai G2F30 instrument (Thermo Fisher Scientific, USA). The Fourier-transform infrared (FT-IR) spectra were measured using an iS10 FT-IR spectrometer (Nicolet Corporation, USA). Atomic force microscopy (AFM) image was obtained on Dimension Icon (Bruker, Germany). To investigate the N- and F-doping status in N, F-CDs, X-ray photoelectron spectroscopy (XPS) was carried out using a 250 Xi instrument (Thermo Fisher Scientific). Confocal microscopic images were obtained using UltraVIEW VoX& IX81 (Olympus, Japan) scanning.

### 2.3. Preparation of N, F-CDs

Ammonium citrate tribasic (243 mg), L-alanine (445 mg), and ammonium fluoride (222 mg) as precursor were added to 10 mL of ultrapure water. The mixture was carefully mixed by ultrasonication (10 min) and then transferred to a polytetrafluoroethylene-lined autoclave (50 mL) and reacted at 160°C for 4 h in an oven. Subsequently, the resulting solution was naturally cooled to room temperature (25°C ± 10°C) and purified using a 0.22 *μ*m end remover filter to remove large particles.

### 2.4. Probe Selectivity and Detection of Hypochlorite

The selectivity of prepared composites was estimated through the addition of various ions and small molecules. Na^+^, H_2_PO_4_^−^, Fe^3+^, ClO_3_^−^, Cl^−^, Ca^2+^, NO_3_^−^, Mn^2+^, HCO_3_^−^, I, Cu^2+^, I^−^, L (+)-ascorbic acid, L-cysteine, K^+^, Ba^2+^, Mg^2+^, Zn^2+^, ClO^−^, S_2_O_8_^2−^, H_2_O_2_, SO_3_^2−^, Cr_2_O_7_^2−^, and MnO_4_^−^ were added to 1.96 mL of N, F-CD solution (0.094 mg/mL). Then, the fluorescence spectrum was measured under an excitation wavelength of 356 nm after 5 min.

The sensitivity of ClO^−^ was investigated in 1.96 mL of N, F-CD solution (0.094 mg/mL). In a typical assay, the fluorescence data of N, F-CDs with different concentrations of ClO^−^ (0.00, 1.00, 6.25, 12.5, 25.0, 50.0, 100, and 600 *μ*M) were investigated. The emission spectrum of the above solution was recorded under excitation wavelength of 356 nm. All measurements were repeated five times.

### 2.5. Real Sample Assays

The feasibility and practicality of the prepared N, F-CDs-based probe were tested by generalizing the detection of different water samples. Lake, tap, and swimming pool water samples were collected from Wanfeng Lake (Xingyi, China), Minzu Normal University of Xingyi (Xingyi, China), and its gymnasium (Xingyi, China), respectively. All raw samples were filtered through a 0.22 *μ*m end remover filter to remove the large particles. The concentration of ClO^−^ in the water samples was detected using the developed probe method. In brief, 40 *μ*L of the water sample was added into the N, F-CD solution (0.094 mg/mL, 1.96 mL), fluorescence intensity was measured at excitation wavelength of 356 nm, and the reliability of the developed method was further assessed via the spiked recovery approach.

### 2.6. Cytotoxicity and Cellular Imaging

Before cellular imaging of RAW 264.7 cells, the MTT assay was used to evaluate the potential cytotoxicity of N, F-CDs for RAW 264.7 cells. The details are as follows: RAW 264.7 cells were incubated and treated with different concentrations (15.6–1000 *μ*g/mL) of N, F-CDs at 37°C for 24 h. Afterwards, the MTT reagent (20 *μ*L, 5 mg/mL) was added to each hole, and the cells were incubated at 37°C for 4 h. Finally, 150 *μ*L dimethyl sulfoxide was added to each hole to dissolve and crystallize the precipitates. Cell survival rate was computed as the ratio of cells in the solution treated with the probe to those in the control group.

To explore the potential application of N, F-CDs, mouse macrophage-like cell line RAW 264.7 was cultured in HyClone with fetal bovine serum (10%, w/w) at 37°C under 5% CO_2_ atmosphere. Then, the cells were transferred to new confocal dishes and divided into five groups. Three groups were incubated with multifarious concentrations (50, 100, and 200 *μ*g/mL) of the N, F-CDs (3 h) and normal saline (4 h) in each well. Meanwhile, the remaining two groups of cells were stimulated by PMA (2 *μ*g/mL) and LPS (100 *μ*g/mL) for 4 h; then, the cell groups were cultivated with N, F-CDs for 3 h to detect the endogenous ClO^−^. The group incubated with 200 *μ*M N, F-CDs was added to each hole and cultivated for 3 h. Subsequently, NaOCl (50 *μ*M) was added for another 4 h to detect the exogenous ClO^−^. Then, phosphate-buffered saline solution (pH = 7.4) was used to wash the cells for three times. The fluorescence of stained cells was observed, and the images were taken in blue channels (370 nm).

## 3. Results and Discussion

### 3.1. Optimization of Preparation Conditions for N, F-CDs

The preparation conditions including reaction time, reaction temperature, and diluted concentration were studied (Figure S1). As shown in Figure S1A, the fluorescence intensity increased with the reaction time from 2–4 h. After 4 h, the fluorescence intensity of N, F-CDs gradually decreased. In consequence, 4 h was selected as the optimal reaction time.  Figure S1B demonstrates that the N, F-CDs illustrate the best fluorescence properties below 160°C. Finally, Figure S1C shows the strongest fluorescence intensity with a raw N, F-CD solution at 80-fold dilution (1.14 mg/mL).

### 3.2. Characterization of N, F-CDs

**Figure 1:**
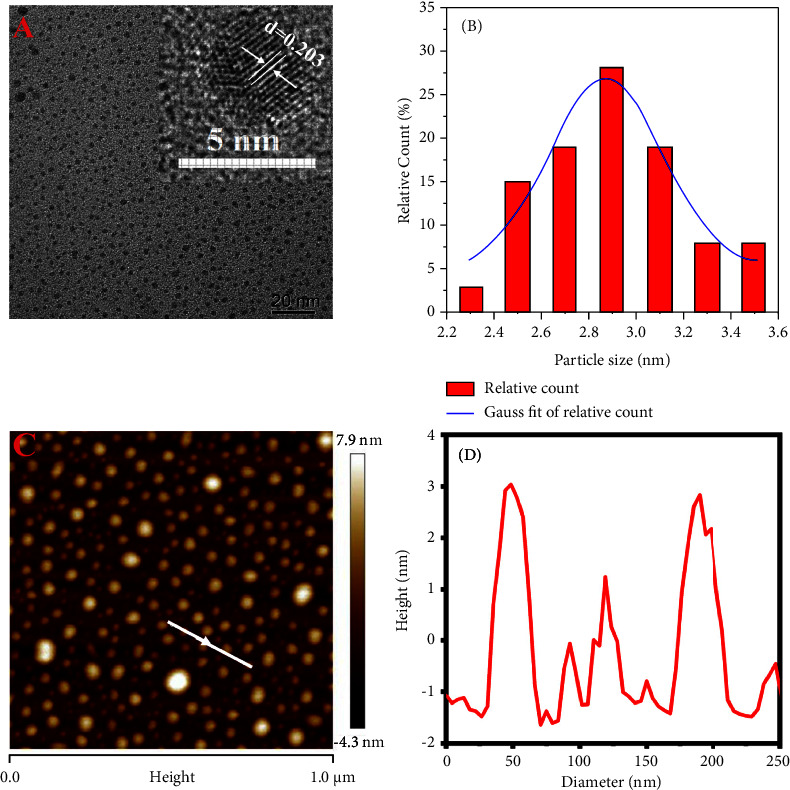
(A) High-resolution transmission electron microscopy (TEM) images of the N, F-CDs (inset). (B) Size distribution. (C) Atomic force microscopy (AFM) images of the nitrogen-fluorine-doped carbon quantum dots (N, F-CDs). (D) Height profile of N, F-CDs.

The morphology of N, F-CDs was characterized by transmission electron microscopy (TEM) and AFM (Figure 1). The TEM images display that the prepared N, F-CDs are spherical composites with an average diameter of 2.9 nm and a narrow particle size distribution of 2.3–3.5 nm (Figure 1(b)). Most particles are amorphous carbon, as can been seen in the high-resolution TEM image. The interplanar spacing of lattice fringes is 0.203 nm, corresponding to the (100) facets of graphitic carbon [21]. As shown in Figures 1(c) and 1(d), the AFM images are consistent with the TEM results, showing an average height of N, F-CDs of approximately 2.9 nm.

To reveal the surface-functional groups of N, F-CDs, the FT-IR and XPS survey spectra were adopted. As shown in Figure S2A, the wide absorption at 3107 cm^−1^ is attributed to the stretching vibrations of O–H [22]. Moreover, the small peaks at 1593 and 1455 cm^−1^ originate from the stretching vibration band of C=C and C–N groups [23], respectively. The peaks at 1014 and 1255 cm^−1^ are consistent with the bending vibrations of the C–O and C–F groups [24], respectively. The characteristic peaks at 1408 and 744 cm^−1^ are attributed to C=N and –NH [25], respectively. Moreover, the XPS survey spectrum, which is further used to investigate the surface state and composition of N, F-CDs, displays the peaks at 284.8, 401.0, 531.9, and 685.6 eV, which are ascribed to C1*s*, N1*s*, O1*s*, and F1*s*, respectively (Figure S2B). The elemental analysis results of N, F-CDs revealed the atomic ratios of C, O, N, and F to be 60.22%, 28.18%, 10.45%, and 1.15%, respectively. This finding indicates considerable doping percentages of F and N.

**Figure 2:**
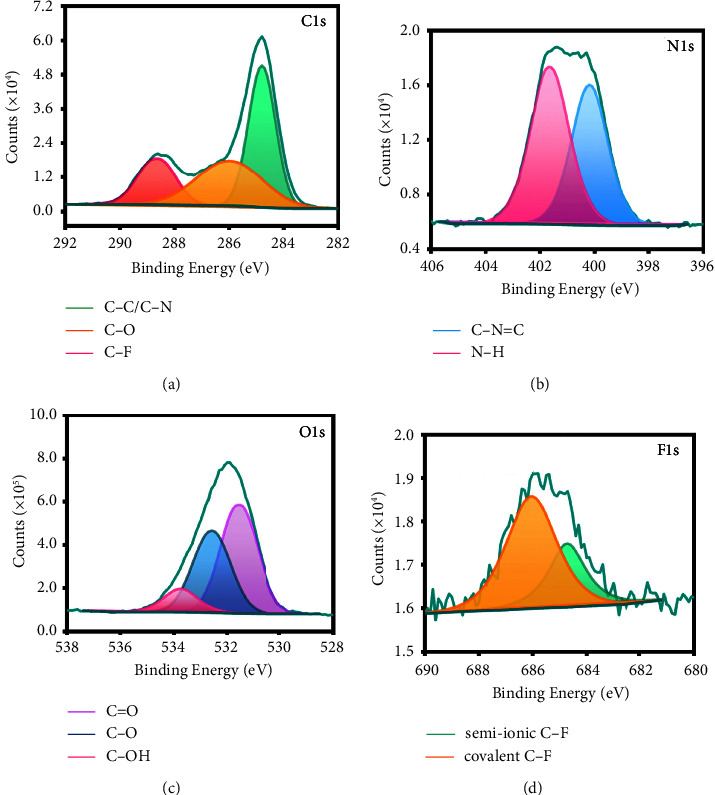
Elemental analysis of N, F-CDs. (a) C1s, (b) N1s, (c) O1s, and (d) F1s high-resolution XPS spectrum.

The high-resolution spectrum of C1*s* (Figure 2(a)) signal exhibits three peaks at 284.8 (C–C/C–N), 285.8 (C–O), and 288.9 eV (C–F). As depicted in Figure 2(b), the two peaks at 399.5 (C–N=C) and 401.6 eV (N–H) appeared in the high-resolution N1*s* spectrum. The O1*s* spectrum (Figure 2(c)) demonstrates three fitted peaks at 531.7 eV, 532.4, and, 533.9 eV, which are attributed to the C=O, C–O, and C–OH groups, respectively. The F1*s* spectrum (Figure 2(d)) peaks at 685.7 and 686.7 eV correspond to the semi-ionic C–F and covalent C–F bonds [24, 26], respectively. The XPS results are consistent with the FT-IR results. Hydrophilic functional groups such as –NH_2_, C–OH, and O=C–OH on the surface of N, F-CDs impart excellent water solubility [27].

### 3.3. Optical Properties of N, F-CDs

**Figure 3:**
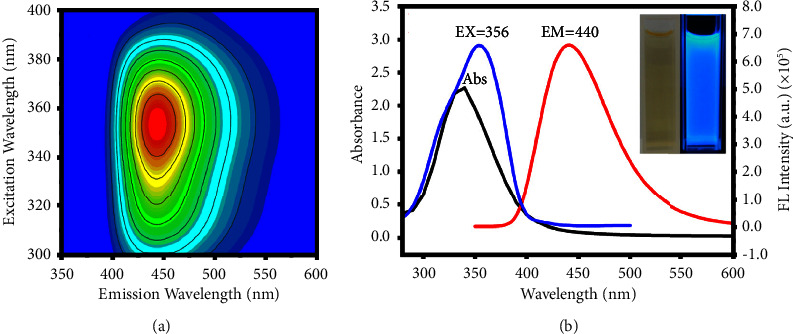
(a) Excitation-emission matrix for the UV-vis absorption spectra of N, F-CDs. (b) UV-vis absorption, fluorescence excitation, and emission spectra of N, F-CDs. Inset shows images of the N, F-CDs under daylight (a) and UV irradiation (b). Abs, absorption; EM, emission; EX, excitation; FL, fluorescence.

The N, F-CDs were distilled with water (1.14 mg/mL), and then their optical properties were explored. It is important to explore the excitation and emission wavelengths of N, F-CDs for their potential applications. The excitation wavelength was approximately 300–390 nm, and the emission band was concentrated at approximately 350–550 nm (Figure 3(a)). The maximum excitation peak was observed at 356 nm, while the maximum emission peak was observed at 440 nm. These findings revealed the typical fluorescence dependence of N, F-CDs between the excitation and emission wavelengths. The aqueous solution of N, F-CDs (Figure 3(b), left inset) presented strong blue fluorescence emission (right) under 356 nm. The UV-vis spectra of N, F-CDs show the characteristic peaks at 340 nm, which can be attributed to the n–*π∗* transition of the N, F-CDs core due to the presence of C=O, C-F, and N-H groups on their surfaces [28].

Fluorescence stability plays a crucial role in quantitative analysis. As shown in Figure S3A, the N, F-CD fluorescence intensity did not change within 24 h, proving the excellent fluorescence stability of N, F-CDs. The photoluminescence response of N, F-CDs was studied by adding NaOH and HCl to adjust the pH to different levels. As illustrated in Figure S3B, the fluorescence intensity of N, F-CDs was closely related to pH; when the pH was 1.0–4.0, the fluorescence intensity increased gradually; within the wide pH range of 4.0–14.0, the fluorescence intensity did not change significantly, which meant that this range was suitable for application. Moreover, quinine sulfate (QY = 54% in 0.1 M H_2_SO_4_ at 360 nm) was used as the standard sample to calculate the fluorescence quantum yield (QY) of N, F-CDs. The average QY of the N, F-CDs in aqueous solution was 26.3% at 24°C ± 4°C.

### 3.4. Fluorescence Measurement of ClO^−^

**Figure 4:**
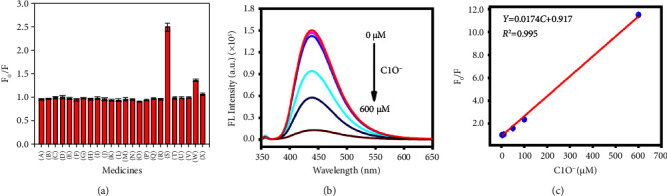
(a) Fluorescence intensities of N, F-CDs alone and of those in the presence of 100 *μ*M of various metal ions and common molecules, including (A) Na^+^; (B) H_2_PO_4_^−^; (C) Fe^3+^; (D) ClO_3_^−^; (E) Cl^−^; (F) Ca^2+^; (G) NO_3_^−^; (H) Mn^2+^; (I) HCO_3_^−^; (J) I; (K) Cu^2+^; (L) I^−^; (M) L (+)-ascorbic acid; (N) L-cysteine; (O) K^+^; (P) Ba^2+^; (Q) Mg^2+^; (R) Zn^2+^; (S) ClO^−^; (T) S_2_O_8_^2−^; (U) H_2_O_2_; (V) SO_3_^2−^; (W) Cr_2_O_7_^2−^; and (X) MnO_4_^−^. (b) Fluorescence intensity of ClO^−^ at different concentrations. (c) Dependence of *F*_0_/*F* on the concentration of ClO^−^.

Thanks to the excellent properties of N, F-CDs, their selectivity was explored in the presence of different ions and small molecules in 100 *μ*M solutions. The species included Na^+^, H_2_PO_4_^−^, Fe^3+^, ClO_3_^−^, Cl^−^, Ca^2+^, NO_3_^−^, Mn^2+^, HCO_3_^−^, I, Cu^2+^, I^−^, L (+)-ascorbic acid, L-cysteine, K^+^, Ba^2+^, Mg^2+^, Zn^2+^, ClO^−^, S_2_O_8_^2−^, H_2_O_2_, SO_3_^2−^, Cr_2_O_7_^2−^, and MnO_4_^−^. The comparison was confirmed based on the relative strength change of *F*_0_/*F*, where *F*_0_ is the fluorescence intensity in the absence of the chaff interferent and *F* is that in the presence of the chaff interferent. For example, Cr_2_O_7_^2−^and MnO_4_^−^produced low quenching, with values that were 1.36, and 1.06 that of ClO^−^, respectively. Evidently, the ClO^−^ ion was quenched with other tested ions and small molecules, indicating that N, F-CDs are selective, highly sensitive, and strongly tolerant in ClO^−^ detection (Figure 4(a)). Furthermore, the fluorescence intensity gradually decreased with increasing concentration of ClO^−^ from 2.5 *μ*M to 600 *μ*M (Figure 4(b)), and the quenched relationship could be quantified using the linear correlation in the following equation:

(1) $$\begin{array}{c} Y=\frac{F_{0}}{F}=0.0174C+0.917\text{,} \end{array}$$

where *F*_0_ and *F* are the fluorescence intensities of N, F-CDs at 356 nm in the absence and presence of ClO^−^, respectively, and *C* is the concentration of ClO^−^. The linear relationship was 2.50–600 *μ*M with a correlation coefficient *R*^2^ of 0.996. The limit of detection was calculated to be (*S*/*N* = 3, *n* = 5) 0.75 *μ*M. The developed probe method clearly demonstrated that N, F-CDs can be used to detect trace ClO^−^ amounts and evidenced their promising applications in environmental and biomedical systems.

### 3.5. Detecting ClO^−^ in Water Samples

To explore the practical applications of N, F-CDs, the developed method was used to detect trace amounts of ClO^−^ in water samples obtained from Wanfeng Lake, tap, and a swimming pool. As shown in Table 1, the recovery ranged from 92.2% to 120%, with relative standard deviations (RSDs) of less than 12%. These results indicate that the N, F-CDs-based probing method was accurate and reliable. Moreover, the proposed method demonstrated that the probe can be used to detect ClO^−^ in different water samples.

### 3.6. Possible Mechanism between N, F-CDs and ClO^−^

**Figure 5:**
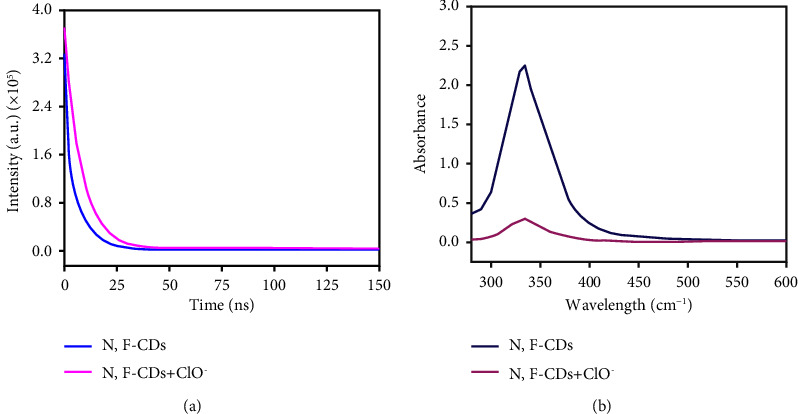
(a) Fluorescence decay times of N, F-CDs and N, F-CDs + 100 *μ*M ClO^−^. (b) UV-vis absorption spectra of N, F-CDs and N, F-CDs + 100 *μ*M ClO^−^.

The possible quenching mechanism between ClO^−^ and N, F-CDs plays a vital role. For an in-depth exploration of the quenching mechanism of ClO^−^ toward N, F-CD fluorescence, the fluorescence lifetimes of N, F-CDs and N, F-CDs+ 100 *μ*M ClO^−^ were evaluated. Their average lifetimes were 8.2 and 7.5 ns, respectively (Figure 5(a)). The lifetime of N, F-CDs+ 100 *μ*M ClO^−^ evidently decreased compared to that of N, F-CDs alone, exhibiting that a dynamic quenching model can be observed. Because static quenching does not shorten the lifetime, fluorescence quenching can be attributed to the dynamic mode. These results were further verified by the UV-vis absorption spectra in Figure 5(b). After adding ClO^−^ to the N, F-CD solution, the absorption intensity markedly decreased at 340 nm. The decrease process revealed that ClO^−^ may selectively oxidate the amino N groups on the surface of N, F-CDs to form new substances with less *π*–*π* and *n*–*π* conjugate systems at 300–600 nm, thereby leading to the fluorescence quenching of N, F-CDs. These results proved that a dynamic quenching mode occurred between N, F-CDs and ClO^−^ [29, 30].

### 3.7. Cytotoxicity Assays and Cell Imaging

To assess the cytotoxicity of N, F-CDs and develop their potential application in bioimaging, traditional MTT assays were employed to test their cytotoxicity in RAW 264.7. As expected, more than 83% of the RAW 264.7 cells were viable after exposure at 500 *μ*g·mL^−1^ (Figure S4). The results exhibit excellent properties of the N, F-CDs, such as low toxicity and excellent biocompatibility, indicating their potential use as biomarkers.

**Figure 6:**
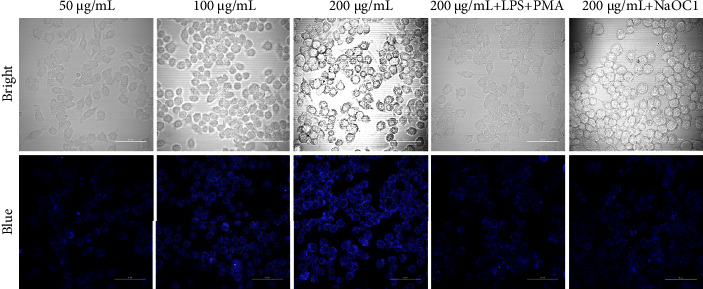
Confocal microscope images of RAW 264.7 cells in N, F-CD solution at an excitation wavelength of 370 nm and emission wavelengths of 430–470 nm.

The practicality and feasibility of N, F-CDs were further evaluated in cell imaging. As shown in Figure 6, the obtained confocal image of RAW 264.7 cells became brighter with increasing concentration of the probe. The overlaid image reveals that N, F-CDs can easily penetrate cell membranes or translocate by endocytosis. Moreover, when the bright N, F-CDs (200 *μ*g/mL) were added with LPS (100 *μ*g/mL) and PMA (2 *μ*g/mL) or NaOCl (50 *μ*M), the intracellular fluorescence confocal intensity of RAW 264.7 cells weakened because of the intracellular presence of trace ClO^−^. Therefore, these experimental results further prove that the N, F-CDs probes can track native ClO^−^ and the fluctuations of endogenous/exogenous ClO^−^ levels in live RAW 264.7 cells.

## 4. Conclusions

Novel N, F-CDs (QY = 26.3%) were successfully synthesized using ammonium citrate tribasic, L-alanine, and ammonium fluoride via a facile, low-cost, ecofriendly hydrothermal approach. The as-prepared N, F-CDs are nanometer-sized (2.9 nm) CDs, exhibiting excellent highly fluorescent stability, low biotoxicity, water solubility, and biocompatibility. Thus, the broad application prospects of N, F-CDs were demonstrated. First, N, F-CDs can function as highly selective and sensitive fluorescent probes for ClO^−^. Second, they can be used for quantitatively detecting ClO^−^ in real water samples, offering a low detection limit of 0.75 *μ*M and broad linear range of 2.50–600 *μ*M. Finally, owing to their low biotoxicity, water solubility, and biocompatibility, N, F-CDs can be used in in vitro imaging. The results show that the probes can not only exhibit cell permeability but can also effectively detect endogenous/exogenous ClO^−^ in living RAW 264.7 cells. This probe is expected to provide a new approach for detecting ClO^−^ in other organelles.

## Figures and Tables

**Table 1:** Determination of ClO^−^ in Wanfeng Lake, tap, and swimming pool water (*n* = 5).

Sample	Concentration (*μ*M)	Spiked concentration (*μ*M)	Found (*μ*M)	Recovery (%)	RSD (%)
Wanfeng Lake	—	5.00	6.01	120	4.1
		50.0	56.5	113	5.5
		100	117	117	2.6

Tap water	5.43 ± 0.59	5.00	11.2	115	11
		10.0	15.1	96.7	3.9

Swimming pool water	—	5.00	6.00	120	12
		50.0	46.0	92.2	5.6
		100	115	115	5.9

## Data Availability

The data used to support the findings of this study are available from the corresponding author upon reasonable request.

## Supplementary Materials

Supplementary Materials Figure S1: optimization of preparation conditions—(A) reaction time, (B) reaction temperature, and (C) diluted concentration. FL, fluorescence. Figure S2: (A) Fourier-transform infrared (FT-IR) spectrum; (B) full X-ray photoelectron spectroscopy (XPS) spectrum. Figure S3: (A) fluorescence stability of time; (B) effect of pH. Figure S4: N, F-CD MTT assay of RAW 264.7 (*n* = 3).
