# Supplementary material for: Preparation of One-Emission Nitrogen-Fluorine-Doped Carbon Quantum Dots and Their Applications in Environmental Water Samples and Living Cells for ClO− Detection and Imaging
Source: J Anal Methods Chem. 2023 Apr 25;2023:7515979. doi: 10.1155/2023/7515979 (PMC10154095; doi:10.1155/2023/7515979)
Supplement: Supplementary Materials — Figure S1: optimization of preparation conditions—(A) reaction time, (B) reaction temperature, and (C) diluted concentration. FL, fluorescence. Figure S2: (A) Fourier-transform infrared (FT-IR) spectrum; (B) full X-ray photoelectron spectroscopy (XPS) spectrum. Figure S3: (A) fluorescence stability of time; (B) effect of pH. Figure S4: N, F-CD MTT assay of RAW 264.7 (n = 3). [file 7515979.f1.docx]

**Preparation of one-emission nitrogen–fluorine-doped carbon quantum dots and their applications in environmental water samples and living cells for** **ClO^-^ detection and imaging**

Qianchun Zhang*, Haijiang Du, Siqi Xie*, Fengling Tian, Xixi Long, Shan Liu, Yun Wu

School of Biology and Chemistry, Key Laboratory for Analytical Science of Food and Environment Pollution of Qian Xi Nan, Minzu Normal University of Xingyi, Xingyi, 562400, P. R. China

E-mail: qianchunzhang@qq.com (QC Zhang)

[siqixie@xynun.edu.cn](mailto:siqixie@xynun.edu.cn) (SQ Xie)

Supporting Information

## 1. Optimization of preparation N, F-CDs conditions


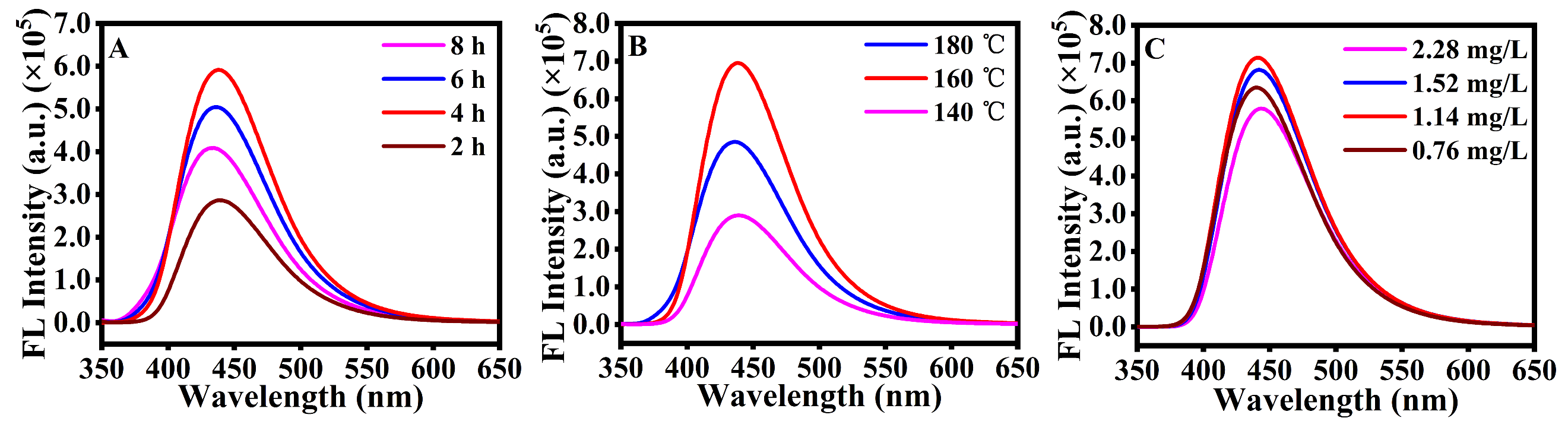


**FIGURE S1.** Optimization of preparation conditions: **(A)** reaction time, **(B)** reaction temperature, and **(C)** diluted concentration. FL, fluorescence.

**2. Characterization of N, F-CDs**


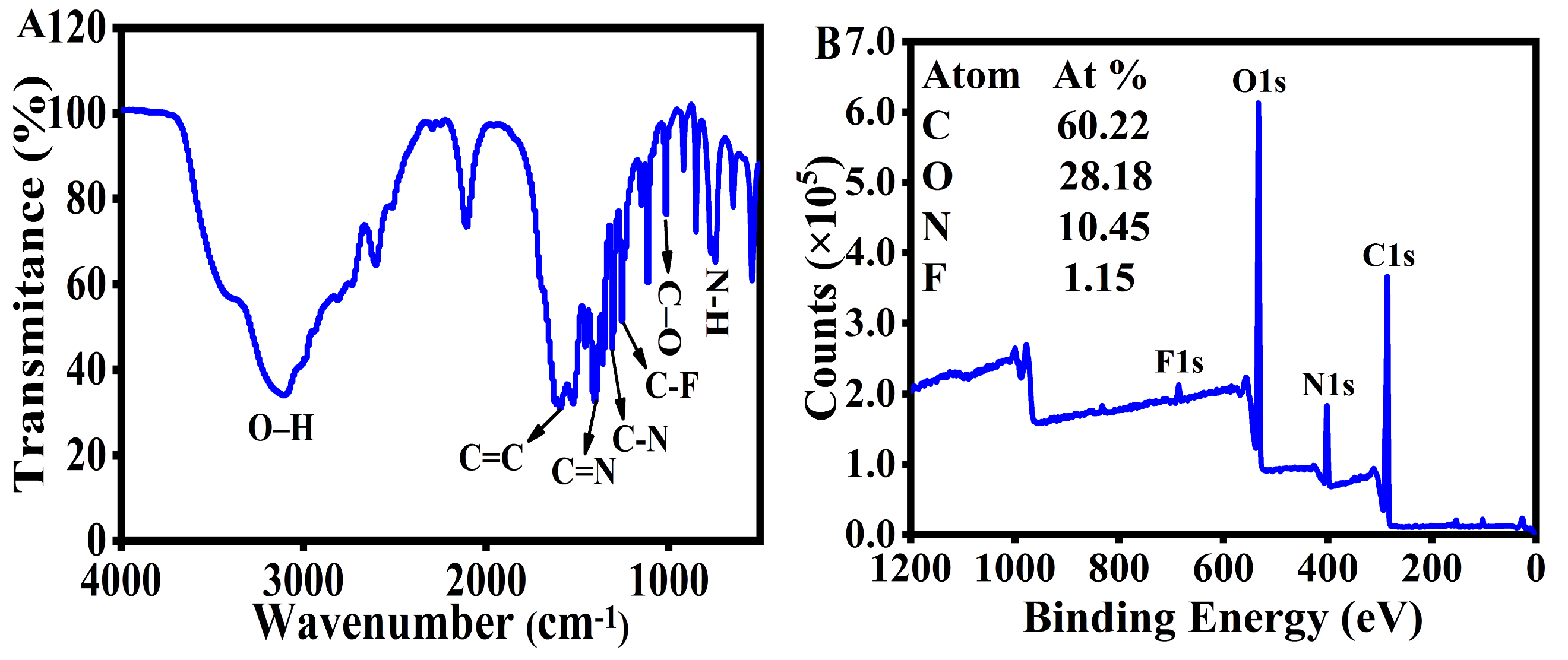


**Figure S2.** **(A)** Fourier-transform infrared (FT-IR) spectrum. **(B)** Full X-ray photoelectron spectroscopy (XPS) spectrum.

**3. Optical properties of N, F-CDs**


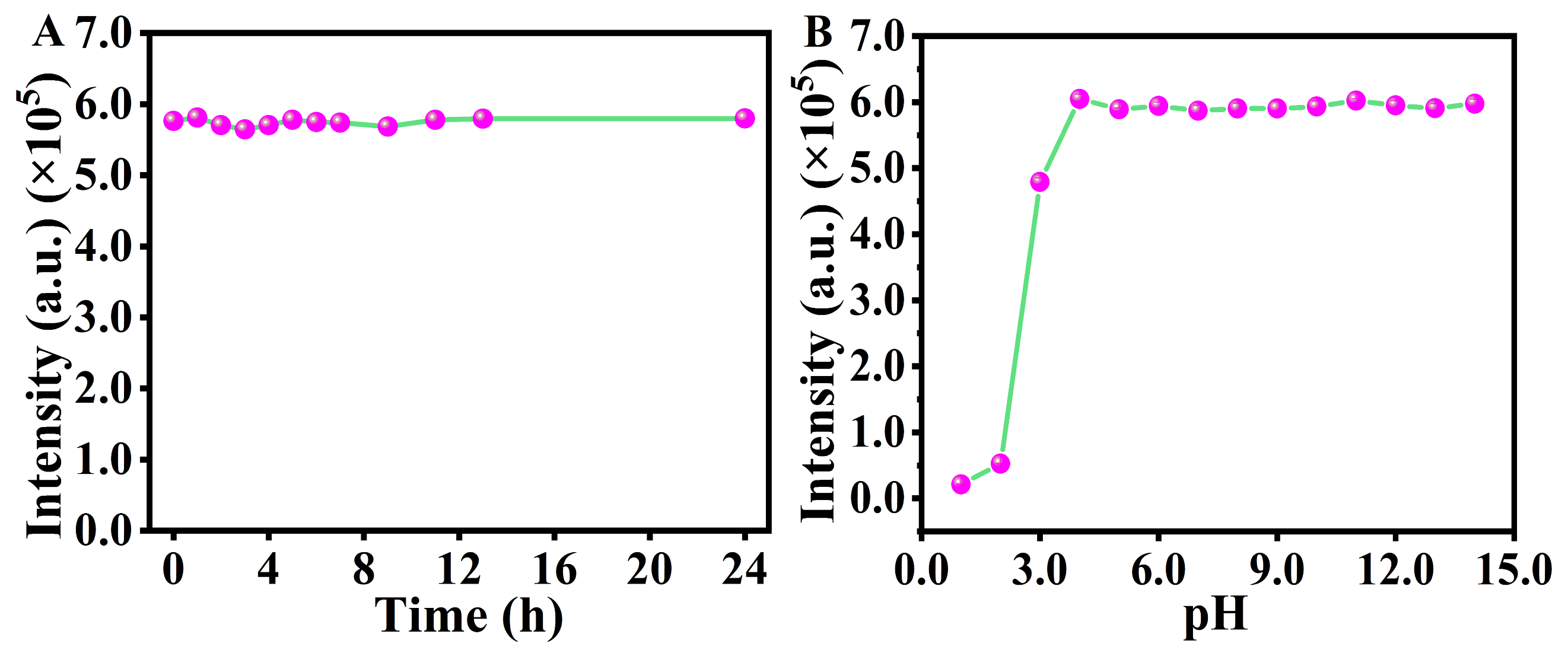


**Figure S3.** **(A)** Fluorescence stability of time. **(B)** Effect of pH.

**4. Cytotoxicity**


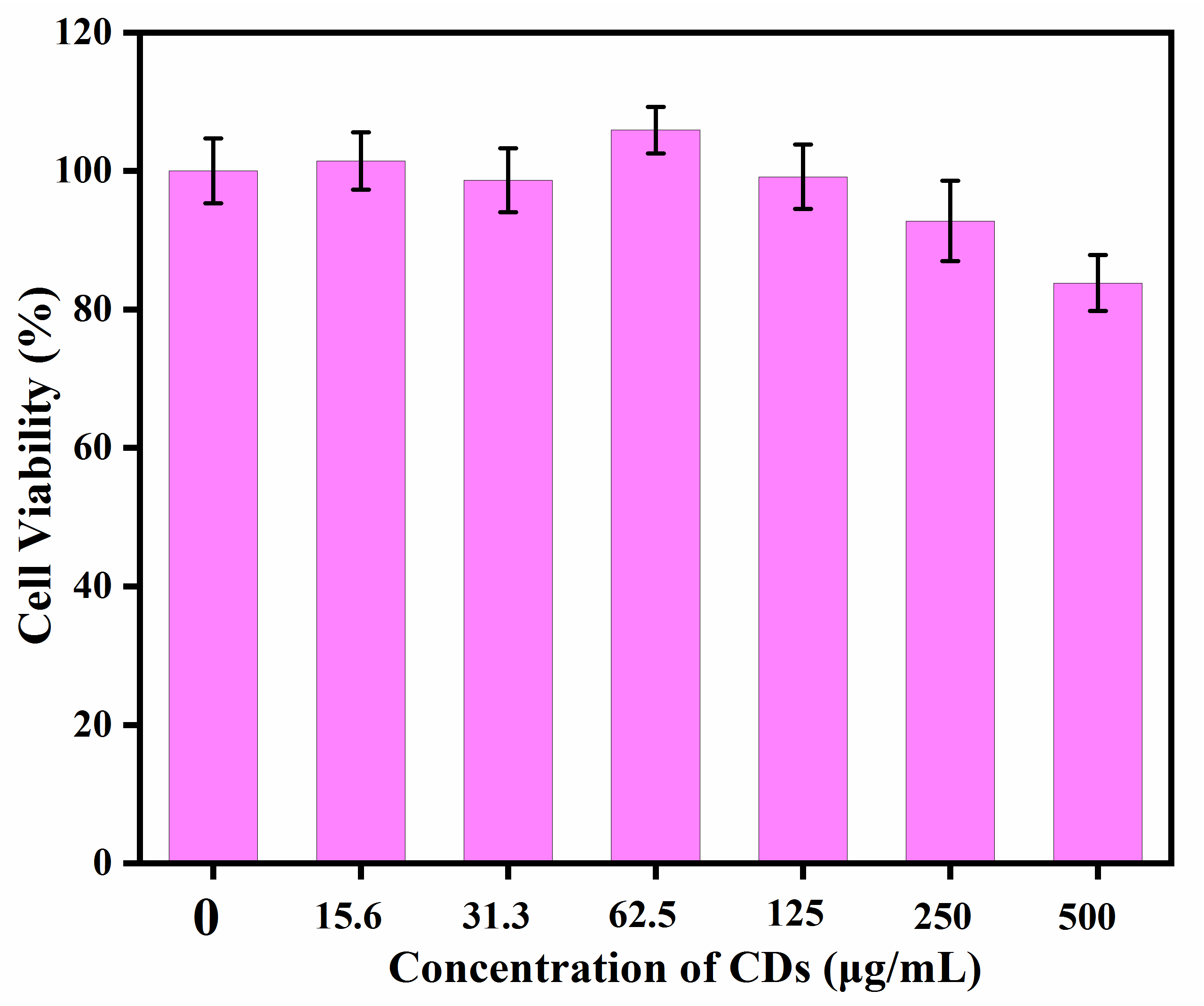


FIGURE S4. N, F-CD MTT assay of RAW 264.7 (*n* = 3)
